# Post-Mortem Inspection of Cattle1Read before the Chicago Veterinary Society, March 12, 1900.

**Published:** 1900-04

**Authors:** James G. Fish

**Affiliations:** Chicago, Ill.


### POST-MORTEM INSPECTION OF CATTLE.[1]

By James G. Fish, M.D.C.,
CHICAGO, ILL.

Instead of directing your attention to the post-mortem evidence of the presence of disease in cattle, I have concluded that it might benefit some here to be more fully informed as to the methods of conducting post-mortem examination now in use at the Union Stock Yards.

In accordance with the plan I shall briefly outline a day's work in post-mortem.

The killing force is divided so that each man has his own particular part of the work to perform.

There will be one knocker, 3 shacklers, 1 to hang off, 2 headers, 1 sticker, 2 switchmen, 1 man dropping cattle, 1 to place cattle on the floor, 5 leg breakers, 2 foot skinners, 6 floormen to skin bellies, 1 brisket sawer, 1 belly washer, 1 man elevating cattle, 1 man skinning hocks, 2 rumpers, 2 shank scrubbers, 1 shank trimmer, 1 shank wiper, 3 fell beaters, 2 backers, 1 back washer, 2 gutters, 3 tail sawers, 4 splitters, 1 tail puller, 4 hide droppers, 1 neck splitter, 43 men wiping and washing cattle before entering the coolers, 60 men doing all around work—*i. e.*, stripping muscle from head, cleaning casings, etc.

This force will kill 114 cattle per hour.

All cattle before reaching the abattoir undergo an ante-mortem inspection.

When the animal has so far advanced as to reach the gutter, the inspector stands behind or a little to one side of him, and notes the contents of the abdomen, the thoracic cavity, and the state of flesh of the animal.

If everything is normal and the animal is in good flesh he passes on to the next carcass, which is only a few feet away. If the conditions are not satisfactory he immediately places a tag of identification on each side of the carcass and a coupon on the internal organs and head and caul fat. They, with the carcass, are put to one side, in a place provided for the same, for future inspection at his leisure.

[1] Read before the Chicago Veterinary Society, March 12, 1900.

As these cattle are killed at the rate of 114 per hour, some may think it is impossible to give a fair and just inspection; but as a man who continually uses a knife becomes an artist at his particular branch of work, so does the inspector become an artist. By long practice and seeing so many healthy animals killed, he becomes expert in detecting the slightest deviation from normal.

Some of you would, no doubt, be surprised at the quickness of a butcher to detect the slightest change from normal; still he may not know what the ailment is.

When a carcass is finally passed on and condemned the inspector has it cut up into suitable pieces for tanking, and goes with it to the tank, and places a seal on the top and bottom of the tank. Steam is then turned on for sufficient time to effectually destroy it for food product.

It may be interesting to notice the number of cattle condemned in Chicago during the last six months and the diseases for which they were condemned, so I have prepared the following list that can be depended on as being approximately correct:

| | Found in animals examined. | Condemned. |
|---|---|---|
| Jaundice | 492 | 1 |
| Uræmia | 600 | 1 |
| Actinomycosis | 55,932 | 115 |
| Metritis | 2,432 | 5 |
| Pneumonia | 11,215 | 23 |
| Bruised carcasses | 25,085 | 52 |
| Leukæmia | 400 | 1 |
| Pleurisy | 6,955 | 13 |
| Dead in pens | 4,651 | 8 |
| Malignant tumors | 1,034 | 2 |
| Enteritis | 3,126 | 6 |
| Bruised parts | 9,116 | 0 |
| Peritonitis | 17,283 | 32 |
| Emaciation | 24,647 | 63 |
| Peritoneal abscesses | 1,322 | 3 |
| Gangrene | 1,790 | 3 |
| Hæmatodes | 9,690 | 21 |
| Cancer | 4,246 | 11 |
| Pyæmia | 2,785 | 5 |
| Septicæmia | 1,768 | 3 |
| Ascites | 1,267 | 2 |
| Pulmonary abscess | 1,075 | 2 |
| Nephritis | 400 | 1 |
| Tuberculosis | 331,887 | 668 |

Making 1041 condemnations for the six months ending December 31, 1899, of which 64.17 per cent. were tuberculous.

Rather than describe any particular disease or the post-mortem evidence of it, I have depended on the discussion to develop the points of greatest interest, and will now pass on to consider a condition not infrequently encountered in cattle, and the cause of much trouble to the inspector.

I refer to *emaciation*, which Gould defines as "loss of the fat and fulness of the flesh of the body." My own idea of emaciation is that it is characterized by extreme poverty of the animal, simple atrophy, in which the fat disappears from the subcutaneous adipose tissue, a diminution takes place in the size of the tissue elements, and the replacement of the once healthy tissue by drops or molecules of an oily nature. When much tissue is affected the change can be readily distinguished with the naked eye by the yellowish-white appearance of the carcass and by a diminution in consistency and elasticity. The largest percentage of emaciated cattle is found in cows formerly used for dairy purposes, that have probably had their milking capacity forced for four or five years. At the end of this period the flow of milk diminishes, and soon the beast is turned out to grass or put on a less nutritious diet, and although allowed to go dry she seems to have lost the power of assimilating food for flesh or fat producing purposes. She becomes thinner each day, and at last is shipped for slaughter where it is sure to bring some sort of a price. The dairyman or any of his neighbors would not be guilty of killing any such animal for food for themselves or families. But he sends it where he thinks it will be overlooked in the great number that daily arrive at the various large markets. I have found it one of my most difficult tasks to condemn a cow for emaciation when there are no other symptoms of disease. The shipper or packer is rare who will gracefully acquiesce in condemnation for such a cause. The question is thus continually forced upon the inspector, What is the degree of emaciation that renders cattle unfit for human food? Anything that will enable him to answer this question to his own and the public's satisfaction he will welcome as a boon, and the discussion that will to any extent develop this point will be of benefit not only to our Society, but to the whole population of this country whose servants we are.

---

Vineland, N. J., following the lead of Bridgeton, is now considering the necessity of establishing a meat-inspection and milk-inspection service. The prevalence of tuberculosis has aroused the people of these towns to the need of better safeguards.

